# Relationship between Preoperative Hemoglobin and Hospital Stays in Patients Receiving Prime Total Knee Arthroplasty

**DOI:** 10.1155/2022/3627688

**Published:** 2022-07-15

**Authors:** Xiao Cai, Peipei Li, Xue Wang, Jing Hu, Lian Wen, Yajing Duan, Zhenzhen Mu, Hong Zhi

**Affiliations:** ^1^Department of Orthopedic Trauma, Honghui Hospital, Xi'an Jiaotong University, Xi'an, China; ^2^Yan'an University, Yan'an, China; ^3^Department of Nursing, Honghui Hospital, Xi'an Jiaotong University, Xi'an, China

## Abstract

Previous evidence has shown that preoperative hemoglobin is related to poor prognosis after primary total knee arthroplasty. Reviewing cohort research was conducted at the third-level academic medical center in Singapore and involved 2,676 patients. Population statistics, complications, preoperative hemoglobin (Hb) levels, length of hospital stay (LOS), and readmission information of thirty days were obtained. Anemia was defined based on the World Health Organization (WHO). LOS extension was with the definition as no less than six days with >1/75 LOS corresponding to the data. According to the study requirements, we finally collected 2273 patients. We plotted the relationship between hemoglobin levels and length of stay. We analyzed 2273 patients, with 140 cases of Hb ≤ 11.0 g/dL, 831 cases of Hb 11.0–12.9 g/dL, and the other 1320 cases of Hb ≥ 13.0 g/dL. The mean age of patients with prolonged LOS (68.4 ± 8.2 years) was higher than that of patients with familiar LOS (65.9 ± 8.0 years). In addition, patients with extended LOS had higher ASA-PS values, a history of cerebrovascular accidents (CVA), diabetes mellitus (DM), and ischemic heart disease (IHD) (*P* < 0.001), repeated surgery within 30 days, HB, and operative time (min) (*P* < 0.01). Variables independently related to increased risk of extended LOS included general anesthesia (GA) (adjusted OR (aOR) 1.4, *P*=0.005, *P*=0.005), CVA (aOR 3.0, *P* < 0.001), DM (aOR 1.4, *P*=0.032), and HB < 11 g/dL. Variables increased LOS included HB ≥ 13 g/dL (aOR 0.4, *P* < 0.001) and Hb 11.0–12.9 g/dL (aOR 0.5, *P*=0.001). Hb was 14 g/dL, and LOS decreased by at least 0.24 days for each 1 g increase in preoperative Hb before the inflection point (95%CI 0.12 to 0.36, *P*=0.0001). Anemia is familiar in patients receiving elective total knee arthroplasty (TKA) in Singapore. Thus, this study describes that the preoperative hemoglobin was associated with length of stay. We found that on the left where HB was 14, length of stay decreased with increased hemoglobin values. We recommend preoperative correction of anemia to determine the diagnosis.

## 1. Introduction

The TKA is a normal surgical therapy for knee degeneration, which is a common disease in the elderly [1]. Meanwhile, TKA is now the most familiar surgical process in the world [2]. Preoperative anemia impacts one-third to two-thirds of sick persons receiving main elected surgery and is related to the increased risk of blood transfusion, hospitalization complications, delayed discharge, and poor recoveries [3, 4]. Recently, Baron et al. [5] studied nearly 40,000 surgical patients in twenty-eight countries in Europe. By surprise, nearly 30% of patients indicated anemia related to the extended hospital stay and increasing death risk in the hospital before operation. In a previous study, led by Lasocki et al. [6], 1534 patients underwent elective internal knee and hip arthroplasty and spinal surgery at 17 European centers. The preoperative prevalence of anemia was 14.1% and over 85.0% at discharge. Meanwhile, lots of research studies have verified the validity of anemia management before surgery [7–13]. Similarly, many research studies on the relationship between the length of hospital stay after joint replacement and preoperative anemia [14–20] have been performed in Western healthcare settings with diverse demographic statistics and possibly different discharge and rehabilitation policies in other parts of the world.

Thus, this study analyzed the effects of preoperative anemia on the length of hospitalization in patients without blood transfusion during the initial total knee arthroplasty.

## 2. Methods

### 2.1. Participants and Data Source

The information for this study uses data from a single-center reviewing study article published by Hairil Rizal Abdullah et al. [21]. The data can be reached via the full dataset used in the analysis that can be downloaded from Dryad public repository at doi:10.5061/dryad.73250. We analyzed electronic medical records of 2,676 patients treated with TKA at Singapore General Hospital between January 2013 and June 2014. The records were obtained from the Singapore General Hospital clinic data system and kept in the Singapore General Hospital company database and analytical system. It combines information from executive management clinic and assistant healthcare institutions. Data from SCM include patient demographic statistics, preoperative complications like smoking, the level of hemoglobin (Hb), personal components of the revised risk cardiac index (RCRI) [22, 23], past records of former CVAs, DM on insulin, and increased preoperative creatinine level >2 mg/dL; ASA-PS value [24]; particulars of surgery like location, time, form of anesthesia, and what day of the week the operation took place [25]; and perioperative blood transfusion and repetitive operations during hospitalization. LOS was counted from the day of permission to the day of discharge to home circumstances. Data on 30-day postdischarge readmission were collected from the clinical information system database, SCM. We diagnostic screened for related readmissions through the internet-classified diseases (10th edition) and further identified the reason for admission by reviewing the electronic medical records of patients. We determined a time window for preoperative hemoglobin levels, at most fourteen days and at least one day before operation. In Singapore General Hospital, many patients are permitted on the day of operation for medical and/or social reasons, and rarely, 1 day earlier.

**Figure 1:**
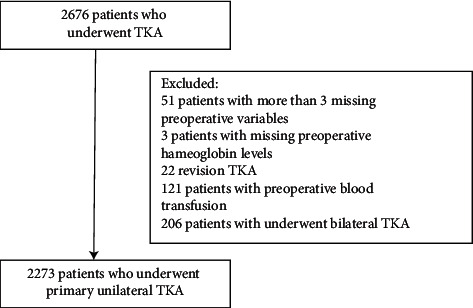
Flowchart shows the derivation of study cohort.

In general, all antiplatelet drugs except aspirin are discontinued before the recommended time of operation. Intraoperative infiltration of the knee with tranexamic acid, intravenous infusion of tranexamic acid, and postoperative drainage into the joint are not standardized. Cell rescue is scarce. After surgery, all patients accepted normal hospital TKA protocol of postoperative care and discharge. Patients received regular physical therapy from the day after surgery, even on the weekends. It contains several procedures to climb, a walking framework to transfer with assistance, and bend the surgically operated knee nearly 90 degrees. After eliminating fifty-one patients with more than 3 variable deletions, 3 patients without preoperative Hb levels, twenty-two patients undergoing revision operation, one hundred and twenty-one patients undergoing preoperative transfusion, and two hundred and six patients undergoing a bilateral operation, we gathered a final analysis of 2273 patients (Figure 1). Due to the small number of data (2.0%), no sensitivity analysis was performed on the missing data.

The primary endpoint was LOS prolongation, with a definition of no less than six days. The cutoff was chosen because it represents >75 centiles for the entire sample. The usage of 75 centimeters to define LOS extension is in accordance with other research studies [26].

### 2.2. Statistical Analysis¶

**Figure 2:**
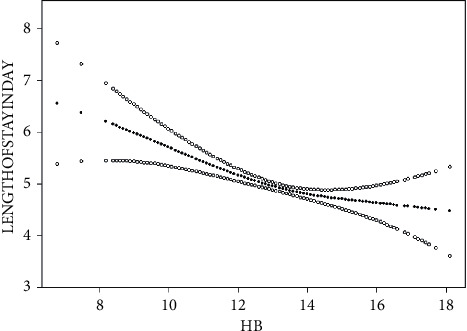
Plot shows the relationship between hemoglobin and length of hospital stay.

Demographic statistics and clinic features of patients were compared (Table 1). We classified age, body mass index (BMI), and duration of surgery by ≤100 min or >100 min (1/75 of > data). A gain in LOS was also determined by using a multivariate logistic regression (Table 2). We applied WHO gender-based definition of anemia seriousness [22]. In the end, we used the data to determine independent predictors of LOS using curve models (Figure 1), while adjusting for demographic factors (Figure 2). Moreover, according to the curve model, we found that the length of hospitalization decreased with increasing Hb levels, but the Hb reached a certain level changed not significantly with increasing hemoglobin levels. Therefore, we propose a threshold effect analysis method based on the curve model to find the inflection point of the curve (Table 2). We apply smooth curve fitting to test whether the independent variables are divided into intervals for the first time. We use piecewise regression (also called piecewise regression), which applies respective line segments to suit every interval. A logarithmic likelihood ratio test was used to compare the single-line (nonpiecewise) model with the piecewise regression model to decide whether the threshold existed. On this basis, the maximum likelihood of the binding point is determined by a two-step recursive approach. Step 1 is to narrow down the inflection point to a 10 percentile range of the independent variable. From 5% to 95% increment by 5%, we test 19 segmented regression models using these 19 percentile points of the independent variable as the inflection point, respectively, to find out which percentile points give the model with the highest likelihood. The precise inflection point was narrowed down to ± 4% percentile of the percentile points, which gives the highest likelihood among the 19 models, called Kmin and Kmax, respectively. Step 2 is to determine the precise inflection point between Kmin and Kmax using the recursive method. The specific method is to first run 3 models with inflection points that equal Q1 (one fourth), Q2 (one half), and Q3 (three fourths) within the range in Kmin and Kmax, respectively, to find out which quartile point gives the model with the highest likelihood among the three models. Then, we narrow down the Kmin and Kmax to the range of ± 25% of the corresponding quartile point. By doing so, we narrow down the range of Kmin and Kmax 50% recursively each time until the specific value of the independent variable was identified, which if used as inflection point will give the segmented regression model the highest likelihood.

## 3. Results

### 3.1. Demographics

There were totally 2273 patients undergoing prime TKA met inclusion. A total of 140 patients were with Hb ≤ 11.0 g/dL, 831 with Hb 11.0–12.9 g/dL, and 1320 with Hb ≥ 13.0 g/dL. In Table 1, the mean age of patients with prolonged LOS (68.4 ± 8.2 years) was higher. There were no evident distinctions in BMI, sex, and anesthesia type in two parts. However, patients with extended LOS wanted to have higher ASA-PS values.

### 3.2. Effects of Hb on LOS¶

The mean LOS in TKA patients was 5.4 days (±4.8 days). The consequences are resembled to former issued LOS ratios [27]. Three hundred and ninety-four patients (17.3%) had LOS over six days (i.e., prolonged LOS). According to a multicomponent study, the variables related to independent increasing risk of extended LOS contain having GA (aOR 1.4, *P*=0.005), previous CVA (aOR 3.0, *P* < 0.001), previous DM (aOR 1.4, *P*=0.032), and HB < 11 g/dL. Variables with decreased LOS contain HB ≥ 13 g/dL (aOR 0.4, *P* < 0.001) and Hb 11.0–12.9 g/dL (aOR 0.5, *P*=0.001). BMI, sex, existence of DM on insulin, former diagnosis of IHD, and creatinine >2 mg/dL were not related to a higher occurrence rate of extended LOS (Table 2).

As mentioned earlier, we performed a multivariate logistic regression analysis of LOS and used gender-based Hb cutoff values. The aOR of HB 11.0–12.9 g/dL was 0.5, and hospital stay was 50% shorter. HB ≥ 13 g/dL extended hospital stay, aOR was 0.4 (0.2, 0.6, *P* < 0.001), and hospital stay was 60% shorter (>6 days).

**Figure 3:**
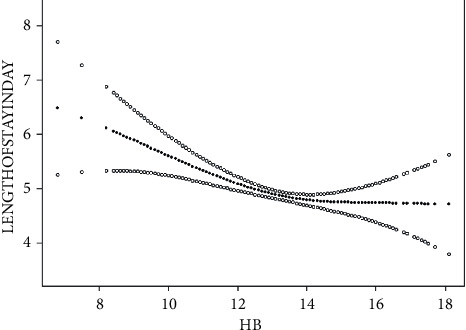
Plot of the relationship between hemoglobin and length of stay after adjustment for the variables.

As shown in Figures 2 and 3, as the Hb protein value changed, it was observed that when Hb is around 14 g/dL, to the left of its value, hospital stay decreases with an increasing hemoglobin value; after adjusting for demographic characteristics such as sex, age, and BMI, the inflection point of the curve changed little. To obtain the accurate inflection point value, we applied the threshold effect analysis based on the curve model to find the inflection point of the curve (Table 3), Hb was 14 g/dL, and for every 1 g of preoperative Hb increase before the inflection point, the LOS decreased by at least 0.24 days.

## 4. Discussion

About one-third of the patients undergoing elective total joint arthroplasty developed anemia without preoperative treatment [4, 5].

Many observed research studies have summarized that preoperative anemia must be thought of as an independent risk element for red blood cell transfusion, latent complications, and postoperative death rate [5, 28, 29]. In this study, with LOS ≤ 6 and HB < 11 g/dL, the prevalence of preoperative moderately severe anemia was 4.6%, LOS > 6, and HB < 11 g/dL was 10.2%. For LOS ≤ 6 and Hb11.0–12.9 g/dL, the prevalence of preoperative mild anemia was 36.2%; for LOS > 6 and Hb11.0–12.9 g/dL, the preoperative prevalence of mild anemia was 41.4%. In our analysis, hemoglobin values were associated with length of stay.

Our results are consistent with former research studies that preoperative anemia independently adds LOS in “fast-track” knee replacement [15] and “conventional” elective prime knee replacement [30]. Therefore, according to current S3 guidelines [31], preoperative diagnosis of anemia should be timely. In this regard, early confirmation of patients with anemia (at least two-four weeks before surgery) is key to surgical preparation. This study now focuses on changes in hemoglobin and length of stay of the TKA patient. Our study indicates in the area of selective orthopedic TKA patient care; on the left where HB was 14 g/dL, length of stay decreased with increased hemoglobin values. Compared with HB < 11 g/dL, aOR 0.5 (0.3, 0.3, *P*=0.001) and days in hospital were 50% less while Hb ≥ 13 g/dL with aOR of 0.4 (0.2, 0.6, *P* < 0.001) of extended LOS and days in hospital were 60% less (>6 days).

The information supports the requirement to cure preoperative anemia as a section of TKA processes in future. Kotze et al. [32] declared resembled success in preoperative anemia management in orthopedic patients. Our research increases to the number of publications from Asia on the negative effect of preoperative anemia on postoperative results of orthopedic and nonorthopedic processes. [33, 34]. The reasons for anemia in orthopedic/trauma patients are various [15, 35–37]. Anemia, at least in these patients, resulted in lack of iron, in a recent study of orthopedic knee patients in Denmark.

Jans et al. [38] indicated over forty percent of patients with anemia showed iron lack. For example, Theusinger et al. [39] have demonstrated the advantages of preoperative anemia management, especially in patients undergoing elective orthopedic surgery. We found that chronic anemia similar to aOR uses the gender definition of anemia, with the definition of mild anemia as 11.0 and 12.9 g/dL in men and 11.0 and 11.9 g/dL in women. Thus, this supports the present proposal that preoperative anemia should be defined at the gender-neutral threshold of <13.0 g/dL for patient blood management purposes [40, 41]. It showed that the curvilinear relationship between hemoglobin value and length of stay, on the left where the hemoglobin value was 14 g/dL and decreased with increasing hemoglobin value, was in line with clinical facts.

Another advantage of our research is that we concluded both subjective tests of clinic risk evaluation: ASA-PS score and RCRI. This study indicated that among the five clinic conditions in the RCRI value range, GA (aOR 1.4, P=0.005), previous CVA (aOR 3.0, P<0.001), and previous DM (aOR 1.4, P=0.032) were associated with increased LOS.

In the end, this study focused on patients who underwent prime TKA, rather than patients who had both hip and knee arthroplasty, which is more common in the literature because we needed a more similar study population. We eliminated revision, perioperative transfusion, and bilateral TKA because these were associated in the literature with an increased need for transfusion and LOS [42, 43]. Our study recruited over a year and a half year. Our recruitment period was resembled to that of another research issued on the theme, which recruited over two years [15].

Because of the observed property of the research, it is difficult to establish a causal relationship between preoperative anemia and negative effects. Furthermore, although our choice of the 75th percentile to define prolonged LOS can be seen as arbitrary truncation in the absence of a widespread definition of extended LOS, similar studies [26] have used the 75th percentile in previous literature. We also do not have data on prevalent intraoperative usage of tranexamic acid that penetrates into the joint, intravenous use of tranexamic acid by anesthesiologists [44–46].

## 5. Conclusions

In conclusion, preoperative hemoglobin was associated with length of stay. We found that on the left where HB was 14, length of stay decreased with increased hemoglobin values. We recommend corrective anemia before operation, containing the usage of a non-sex-based Hb cutoff to determine the diagnosis.

## Figures and Tables

**Table 1:** Demographics of patients with normal LOS (≤6 days) versus those with prolonged LOS (>6 days) after primary unilateral TKR.

Variable		LOS ≤ 6 *N* = 1879	LOS > 6 *N* = 394	*P* value
Patient demographics				
Age		65.9 ± 8.0	68.4 ± 8.2	<0.001
Race (*n*%)				0.023
	Chinese	1585 (84.4%)	329 (83.5%)	
	Malay	143 (7.6%)	20 (5.1%)	
	Indian	101 (5.4%)	29 (7.4%)	
	Others	50(2.8%)	16 (4.1%)	
Gender				0.310
	Female	1414 (75.3%)	306 (77.7%)	
	Male	465 (24.7%)	88 (22.3%)	
BMI				0.158
	≤25	543 (29.4%)	117 (31.0%)	
	>25, ≤30	794 (42.9%)	163 (43.1%)	
	>30, ≤35	393 (21.3%)	65 (17.2%)	
	>35	119 (6.4%)	33 (8.7%)	
Details of operation				
Operation duration (min)		79.6 ± 21.6	83.7 ± 25.9	0.015
Type of anesthesia				0.102
	RA	1254 (66.7%)	242 (61.4%)	
	GA	604 (32.1%)	146 (37.1%)	
	GA RA	19 (1.0%)	5 (1.3%)	
	GA LA	1 (0.1%)	0 (0.0%)	
	GA other	1 (0.1%)	0 (0.0%)	
	RA other	0 (0.0%)	1 (0.3%)	
Day of week of op				<0.001
	Thursday	459 (24.4%)	54 (13.7%)	
	Tuesday	408 (21.7%)	102 (25.9%)	
	Wednesday	320 (17.0%)	74 (18.8%)	
	Monday	302 (16.1%)	77 (19.5%)	
	Friday	288 (15.3%)	72 (18.3%)	
	Saturday	102 (5.4%)	15 (3.8%)	
Patient comorbidities				
Smoking				0.897
	No	1697 (90.3%)	355 (90.1%)	
	Yes	182 (9.7%)	39 (9.9%)	
DM				0.001
	No	1553 (82.7%)	298 (75.6%)	
	Yes	326 (17.3%)	96 (24.4%)	
DM on insulin				
	No	1398 (74.4%)	300 (76.1%)	0.648
	Null	453 (24.1%)	87 (22.1%)	
	Yes	28 (1.5%)	7 (1.8%)	
IHD				0.025
	No	1792 (95.4%)	365 (92.6%)	
	Yes	87 (4.6%)	29 (7.4%)	
CCF				0.583
	No	1869 (99.5%)	391 (99.2%)	
	Yes	10 (0.5%)	3 (0.8%)	
CVA				<0.001
	No	1854 (98.7%)	375 (95.2%)	
	Yes	25 (1.3%)	19 (4.8%)	
Creatinine >2 mg/dL				0.352
	No	1671 (88.9%)	344 (87.3%)	
	Null	199 (10.6%)	46 (11.7%)	
	Yes	9 (0.5%)	4 (1.0%)	
Repeat op within 30 days				<0.001
	No	1870 (99.7%)	387 (98.2%)	
	Yes	5 (0.3%)	7 (1.8%)	
HB categorical				<0.001
	<11	87 (4.6%)	40 (10.2%)	
	≥11, <13	681 (36.2%)	163(41.4%)	
	≥13	1111 (59.1%)	191 (48.5%)	
ASA-PS				0.013
	1	136 (7.2%)	24 (6.1%)	
	2	1650 (87.8%)	336 (85.3%)	
	3	93 (4.9%)	34 (8.6%)	

ASA-PS, American Society of Anesthesiologist Physical Status; CCF, congestive cardiac failure; CVA, cerebrovascular accidents; DM, diabetes mellitus; GA, general anesthesia; IHD, ischemic heart disease; LOS, length of stay; RA, regional anesthesia; TKR, total knee replacement. ASA-PS, American Society of Anesthesiologist Physical Status; CCF, congestive cardiac failure; CVA, cerebrovascular accidents; DM, diabetes mellitus; GA, general anesthesia; IHD, ischemic heart disease; LOS, length of stay; RA, regional anesthesia; REF, reference.

**Table 2:** Variables that predict increased LOS in hospital after primary unilateral total knee replacement, based on univariate and multivariate analyses.

Variable	OR (95%CI)	*P* value	OR (95%CI)	*P* value
Age	1.0 (1.0 to 1.1)	<0.001	1.0 (1.0 to 1.1)	<0.001
BMI				
<25	REF		REF	
≥25, <30	1.0 (0.7 to 1.3)	0.857	1.0 (0.8 to 1.4)	0.797
≥30, <35	0.8 (0.6 to 1.1)	0.168	0.9 (0.6 to 1.3)	0.507
≥35	1.3 (0.8 to 2.0)	0.292	1.6 (1.0 to 2.7)	0.068
Race				
Chinese	REF		REF	
Malay	0.7 (0.4 to 1.1)	0.109	0.6 (0.3 to 1.1)	0.082
Indian	1.4 (0.9 to 2.1)	0.139	1.4 (0.9 to 2.2)	0.167
Gender				
Female	REF		REF	
Male	0.9 (0.7 to 1.1)	0.311	0.9 (0.7 to 1.3)	0.684
Type of anesthesia				
RA	REF		REF	
GA	1.3 (1.0 to 1.6)	0.052	1.4 (1.1 to 1.8)	0.005
GA RA	1.4 (0.5 to 3.7)	0.541	1.5 (0.5 to 4.1)	0.479
Operation duration (min)	1.0 (1.0 to 1.0)	<0.001	1.0 (1.0 to 1.0)	0.005
Day of week of op				
Thursday	REF		REF	
Tuesday	2.1 (1.5 to 3.0)	<0.001	2.4 (1.7 to 3.5)	<0.001
Wednesday	2.0 (1.3 to 2.9)	<0.001	2.0 (1.3 to 3.0)	<0.001
Monday	2.2 (1.5 to 3.2)	<0.001	2.1 (1.4 to 3.2)	<0.001
Friday	2.1 (1.4 to 3.1)	<0.001	1.9 (1.3 to 2.9)	0.001
Saturday	1.2 (0.7 to 2.3)	0.474	1.4 (0.7 to 2.7)	0.286
HB				
<11	REF		REF	REF
≥11, <13	0.5 (0.3, to 0.8)	0.002	0.5 (0.3 to 0.8)	0.001
≥13	0.4 (0.2, 0.6)	<0.001	0.4 (0.2, 0.6)	<0.001
Smoking				
No	REF		REF	
Yes	1.0 (0.7 to 1.5)	0.897	1.1 (0.7 to 1.7)	0.725
DM				
No	REF		REF	
Yes	1.5 (1.2 to 2.0)	0.001	1.4 (1.0 to 1.8)	0.032
IHD				
No	REF		REF	
Yes	1.6 (1.1 to 2.5)	0.026	1.3 (0.8 to 2.2)	0.238
CCF				
No	REF		REF	
Yes	1.4 (0.4 to 5.2)	0.585	1.4 (0.3 to 5.4)	0.671
CVA				
No	REF		REF	
Yes	3.8 (2.0 to 6.9)	<0.001	3.0 (1.6 to 5.9)	<0.001
Creatinine >2 mg/dL				
No	REF		REF	
Yes	2.2 (0.7 to 7.1)	0.202	1.9 (0.5 to 6.9)	0.313
DM on insulin				
No	REF		REF	
Yes	1.2 (0.5 to 2.7)	0.721	1.1 (0.5 to 2.8)	0.782

**Table 3:** Threshold effect analysis between hemoglobin and length of stay for exposure: HB.

Outcome	Length of stay	*P* value
Inflection point (K)	14	
<*K* piecewise 1	−0.24 (0.36, 0.12)	<0.0001
>*K* piecewise 2	0.15 (0.12, 0.41)	0.2771
Log-likelihood ratio test		0.020

Data in the table: *β* (95% CI) *P* value/OR (95% CI) *P* value.

## Data Availability

The datasets used and analyzed during the current study are available from the corresponding author on reasonable request.
